# Autonomic Test by EZSCAN in the Screening for Prediabetes and Diabetes

**DOI:** 10.1371/journal.pone.0056480

**Published:** 2013-02-12

**Authors:** Zhi Yang, Baihui Xu, Jieli Lu, Xiaoguang Tian, Mian Li, Kan Sun, Fei Huang, Yu Liu, Min Xu, Yufang Bi, Weiqing Wang

**Affiliations:** 1 Key Laboratory for Endocrine and Metabolic Diseases of Ministry of Health, Rui-Jin Hospital, Shanghai Jiao Tong University School of Medicine, E-Institute of Shanghai Universities, Shanghai, People's Republic of China; 2 Shanghai Clinical Center for Endocrine and Metabolic Diseases, Shanghai Institute of Endocrine and Metabolic Diseases, Department of Endocrinology and Metabolism, Rui-Jin Hospital, Shanghai Jiao Tong University School of Medicine, Shanghai, People's Republic of China; Institute for Nanotechnology and Stem Cell Biology, United States of America

## Abstract

**Background:**

Autonomic neuropathy is common in diabetics and may occur in prediabetes. A new and noninvasive autonomic test-EZSCAN evaluates sudomotor function precisely. No generally accepted EZSCAN thresholds to screen for prediabetes and diabetes have been defined.

**Methodology and Principal Findings:**

Cross-sectional study of 5, 824 Chinese adults aged 40 and older was conducted in Shanghai, China. We used EZSCAN to evaluate autonomic function in different glucose status and screen for prediabetes and diabetes. The prevalence of prediabetes and diabetes were 21.9% and 17.5% respectively. Compared with the lowest quintile, the highest quintile of EZSCAN value had odds ratios for having dysglycemia (prediabetes or diabetes) of 2.08 (95% CI 1.67–2.58) in total population, 2.89 (95% CI 2.06–4.05) in men and 1.70 (95% CI 1.28–2.25) in women after adjustment for confounding factors. EZSCAN value improved the areas under ROC curve for detection of dysglycemia or diabetes beyond the contribution of conventional risk factors by 0.8% and 12.9%. The cut-off point of EZSCAN value higher than 30% provided reasonable sensitivities (70.3–83.7%) to detect dysglycemia not only in total population regardless of sex but also in individuals with high risk of developing diabetes.

**Conclusions and Significance:**

EZSCAN value higher than 30% indicate an increased risk of prevalent prediabetes and diabetes, suggesting that subjects with EZSCAN ≥30% should be further evaluated by oral glucose tolerance test. The improvement of EZSCAN for diabetes detection was still of limited clinical relevance. Thus the clinical application value of EZSCAN is needed to be explored in future studies.

## Introduction

Type 2 diabetes is now recognized as an immense and growing public health challenge worldwide. Globally, diabetes affected an estimated 366 million adults in 2011-a figure predicted to rise to 552 million by 2030 [1]. In China, the prevalence of total prediabetes and diabetes were 15.5% and 9.7%, respectively in 2007 [2]. Patients with type 2 diabetes are at high risk of mortality as a result of myocardial infarction, stroke, retinopathy, nephropathy, neuropathy, and etc. Early diagnosis of prediabtes and diabetes can result in appropriate interventions which can reduce the incidence of negative complications. Therefore, it is of paramount importance to adopt simple and inexpensive methods in screening for high risk individuals.

Autonomic neuropathy is common in diabetes population. It may be either clinically evident or subclinical with dysfunctions of one or more systems, including cardiovascular, gastrointestinal, genitourinary systems and sudomotor or ocular functions. Among these involved systems, sudomotor dysfunction was regard as the initial component of autonomic neuropathy [3]. Assessment of sudomotor function contributes to the detection of autonomic dysfunction in diabetics [4], [5].

The available techniques for assessing sudomotor function include the quantitative sudomotor axon reflex test (QSART), the thermoregulatory sweat test, silicone impressions, the quantitative direct and indirect reflex test (QDIRT) and the Sympathetic skin response (SSR) [6]. QSART is capable of detecting distal small fiber polyneuropathy with a sensitivity of more than 75% and may be considered as the reference method. However, it requires a high level of clinical expertise to perform and is too time-consuming to practice in epidemiology researches.

A new autonomic test-EZSCAN (Impeto Medical, Pairs, France), which can perform a precise evaluation of sudomotor function, is an alternative to QSART because of its noninvasiveness, time-saving and easiness of practice [7]. Several small sample size studies [7], [8], [9] assessed this device and recommended it for the screening of diabetes. However, it is indispensable to confirm the usefulness of EZSCAN in detecting diabetes by large scale population.

Besides, the association between neuropathy and prediabetes remains controversial. Autonomic dysfunction may have existed for a long time before the diagnosis of diabetes [10]. Thus, association between autonomic function and different glucose status were explored and the optimal EZSCAN cut-off points to screening for subjects at high risk of prediabetes and diabetes were suggested in the present population-based study involving a total of 5, 824 individuals aged 40 years and older.

## Methods

We reported the present study in accordance with the Standards for the Reporting of Diagnostic accuracy studies (STARD) statement [11].

### Ethics statement

The study protocol was approved by the Institutional Review Board of the Rui-jin Hospital affiliated to Shanghai Jiao-Tong University School of Medicine. The written informed consent was obtained from each participant.

### Study population

The present EZSCAN study was a part of our community-based program investigating epidemiology of metabolic diseases and their risk factors, which was conducted in two nearby communities in the same district of Shanghai, China, from March to August 2010. During the recruiting phase, 10, 569 inhabitants, aged 40 yr and older, were invited by telephone or door-by-door visits to participate in this program. From them, 10, 375 (98.2%) women and men agreed to take part. Among total participants, the first 6, 000 subjects during March to June 2010 were consecutively arranged the EZSCAN measurement. Recommend by the Impeto Medical, 113 individuals who met the exclusion criteria: 1) those with pacemaker or defibrillator; 2) subjects with changeable dynamic electrocardiogram; 3) those with alcohol consumption or beta-blockers before the test; 4) individuals with amputation, did not perform- this test [12]. Moreover, 63 subjects with unknown information about self-reported previous history of diabetes or uses of antidiabetic medications were further excluded due to the inability to diagnose dysglycemia. Eventually, 5, 824 subjects aged 40 yr and older (age range, 40–92 yr) were included in the final analysis.

### Data Collection

A standard questionnaire was administered by trained staff to obtain information on demographic characteristics, medical history and lifestyle risk factors. Anthropometric measurements including body weight, height and waist circumference were done using standardized procedures. Body mass index (BMI) was calculated as body weight in kilograms divided by body height squared in meters (kg/m^2^). Waist circumference was measured at the level of the umbilicus with participants in the standing position. Three sitting blood pressure measurements taken consecutively with 1-minute intervals using an automated electronic device (OMRON Model HEM-752 FUZZY, Omron Company, Dalian, China) were averaged for analysis. All participants were undertaken a 75-g oral glucose tolerance test (OGTT) performed by a nurse, and blood samples were collected at 0 and 2 hours to test the fasting blood glucose (FBG) and postprandial blood glucose (PBG) respectively by 2 specialized nurses. The fasting blood sample was also used for other biochemical measurements.

### Laboratory measurements

Plasma glucose was measured using the glucose oxidase method on an autoanalyser (Modular P800, Roche, Basel, Switzerland). Serum total cholesterol (TC), triglyceride (TG), low-density lipoprotein cholesterol (LDL-C) and high-density lipoprotein cholesterol (HDL-C) were measured using chemiluminescence methods on the autoanalyser (Modular E170, Roche, Basel, Switzerland). The HbA1c level was measured by high-performance liquid chromatography (BIO-RAD Company, USA).

### Measurement of sudomotor function

Sudomotor function was evaluated by EZSCAN device from March to June, 2010. The EZSCAN device is designed to perform a precise evaluation of sweat gland function based on measurement of sweat chloride concentrations using reverse iontophoresis and chronoamperometry [13], [14]. Reverse iontophoresis extracts ions from the sweat which is secreted by sympathetic controlled eccrine glands. The extracted sweat creates a current when it encounters specific sensors such as Ni electrodes. The current produced is proportional to the chloride concentration that reacts specifically with the Ni electrodes at low direct-current (DC) stimuli. A time/ampere curve is recorded for each derivation. The conductance is the ratio between current generated and the constant DC stimulus. Basis on the electrochemical skin conductance of head, hands and feet as well as some demographic data, including sex, age, height, weight and systolic blood pressure, the EZSCAN value, which ranges from 0 to 100%, is calculated by the Impeto Medical algorithm and displays on a standard personal computer. Higher reading indicates higher prevalence of sudomotor dysfunction. This measurement was proved to be reproducible in various conditions with low influence of usual physiological variations [15]. The device was calibrated every morning before measurements in accordance with the manufacturer's recommendations. Subjects were measured by one of two fixed machines which were operated by two trained staff. The operators didn't know the glucose metabolism status of subjects. No adverse events occurred during the test.

### Assessment of glucose tolerance status

In accordance with the 2006 World Health Organization (WHO) diagnostic criteria, diabetes was defined as 1) FBG ≥7.0 mmol/L, or 2) PBG ≥11.1 mmol/L, or 3) self-reported previous diagnosis of diabetes by physicians or use of antidiabetic medications. Prediabetes was defined as 1) FBG ranged from 6.1 mmol/L to 6.9 mmol/L, and/or 2) PBG ranged from 7.8 mmol/L to 11.0 mmol/L. FBG<6.1 mmol/L and PBG<7.8 mmol/L were defined as normal glucose tolerance (NGT). Dysglycemia was defined as prediabetes or diabetes.

### Statistical analyses

SAS 9.2 (SAS Institute, Cary, NC) was used for all statistical analyses. All continuous variables were presented as means ± standard deviation (SD) or medians (interquartile ranges). FBG, PBG, HbA1c, TC, TG, HDL-C and EZSCAN value were logarithmically transformed to achieve a normal distribution. All categorical variables were presented as numbers (proportions). Comparisons of means and proportions were performed with ANOVA test and Chi-squared test. Homogeneity of groups was determined when the means compared by the Student-Newman-Keuls method showed significant differences.

Odds ratios (ORs) and 95% confidence intervals (CIs) were examined by logistic regression analyses. To measure the performance of EZSCAN value for detecting dysglycemia, we used the receiver operating characteristic curve (ROC) to calculate corresponding areas under the curve (AUC) and compared the AUCs by logistic regression models incorporating some conventional risk factors [age, BMI, family history of diabetes, women who delivered a giant baby or who were diagnosed with gestational diabetes mellitus (GDM), history of cardiovascular disease (CVD), systolic blood pressure, diastolic blood pressure, HDL-C and TG] with and without EZSCAN value [16], [17]. In order to obtain a better assessment of the prediction power of the EZSCAN test, we used the optimal operating point with setting the minimum sensitivity of 70% for which we did not want sensitivity to fall below [18]. Besides, we calculated the sensitivities of EZSCAN value 25%, 50% and 75% (each one-quarter increase from 0 to 100%) according to the recommended criterion which was formed by the Impeto Medical algorithm. A *P* value of less than 0.05 was considered to be statistically significant.

## Results

### Characteristics of the population stratified by glucose status

The present data included 5, 824 participants (40.3% men) aged more than 40 (58.3±10.0) years during March to June 2010. General characteristics of the study population stratified by glucose status are shown in Table 1. The overall prevalence of prediabetes and diabetes was 21.9% and 17.5%. Participants with dysglycemia were older, more likely to be of higher BMI, waist circumference, blood pressure, and had significantly higher levels of TG, TC, LDL-C, while lower levels of HDL-C, compared with NGT group (all *P* values<0.0001). The medians (interquartile ranges) of EZSCAN values were 31% (26–50%) in NGT group, 44% (28–52%) in prediabetes group and 51% (32–55%) in diabetes group, respectively (*P* for trend <0.0001).

**Table 1:** Characteristics of study population.

Characteristic	NGT (0)	Prediabetes (1)	Diabetes (2)	*P* value	Homogeneity of groups	*P* for trend
n (%)	3530 (60.6)	1277 (21.9)	1017 (17.5)	-	-	-
Men, n (%)	1419 (40.2)	474 (37.1)	450 (44.6)	0.0015	-	0.11
Age (years)	56.6±9.9	60.4±9.7	61.5±9.8	<0.0001	(0) (1) (2)	<0.0001
BMI (kg/m^2^)	24.6±3.1	25.6±3.3	26.2±3.5	<0.0001	(0) (1) (2)	<0.0001
Waist circumference (cm)	81.0±8.8	83. 8±8.7	86. 9±9.2	<0.0001	(0) (1) (2)	<0.0001
Current smoker, n (%)	837 (24.3)	205 (16.6)	219 (22.2)	<0.0001	-	0.0031
Current drinker, n (%)	383 (11.1)	132 (10.6)	110 (11.2)	0.87	-	0.91
Systolic blood pressure (mmHg)	137±19	145±19	150±20	<0.0001	(0) (1) (2)	<0.0001
Diastolic blood pressure (mmHg)	82±10	84±10	84±11	<0.0001	(0) (1, 2)	<0.0001
TC (mmol/L)	5.15 (4.54–5.78)	5.41 (4.75–6.09)	5.42 (4.82–6.17)	<0.0001	(0) (1, 2)	<0.0001
TG (mmol/L)	1.23 (0.89–1.74)	1.58 (1.08–2.16)	1.65 (1.19–2.34)	<0.0001	(0) (1) (2)	<0.0001
LDL-C (mmol/L)	3.07±0.82	3.25±0.87	3.29±0.94	<0.0001	(0) (1, 2)	<0.0001
HDL-C (mmol/L)	1.33 (1.11–1.58)	1.27 (1.08–1.51)	1.23 (1.06–1.47)	<0.0001	(0) (1) (2)	<0.0001
FBG (mmol/L)	5.0 (4.6–5.3)	5.4 (5.0–6.0)	7.1 (6.1–8.3)	<0.0001	(0) (1) (2)	<0.0001
PBG (mmol/L)	5.9 (5.0–6.7)	8.7 (8.1–9.5)	14.3 (11.9–17.7)	<0.0001	(0) (1) (2)	<0.0001
HbA1c (%)	5.5 (5.3–5.7)	5.7 (5.5–6.0)	6.6 (6.0–7.6)	<0.0001	(0) (1) (2)	<0.0001
EZSCAN value (%)	31 (26–50)	44 (28–52)	51 (32–55)	<0.0001	(0) (1) (2)	<0.0001

Data were means ± SD or medians (interquartile ranges) for skewed variables or numbers (proportions) for categorical variables. *P* values were for the ANOVA or Chi-squared tests across three groups. Statistic differences among three groups settle in various parentheses which show in Homogeneity of groups. *P* for trend was calculated from Chi-squared tests for categorical variables and linear regression analyses for continuous variables.

Abbreviations: BMI, body mass index; TC, total cholesterol; TG, triglycerides; LDL-C, low-density lipoprotein cholesterol; HDL-C, high-density lipoprotein cholesterol; FBG, fasting blood glucose; PBG, postprandial blood glucose.

### The association between EZSCAN value and dysglycemia

The quintile ranges for EZSCAN value in total participants were 0–25%, 26%–29%, 30%–46%, 47%–53% and 54%–100%. The sex-specific quintile ranges were 0–25%, 26–28%, 29–44%, 45–52%, 53–100% in men and 0–25%, 26–30%, 31–46%, 47–53%, 54–100% in women. Compared with the lowest quintile, multivariate-adjusted ORs of having prediabetes, diabetes or dysglycemia by each quintile increment of EZSCAN value are presented in Table 2.

**Table 2:** Association between EZSCAN value and risks of having dysglycemia through quintile increment of EZSCAN value.

	Adjusted OR (95% CI)^a^					
	Q1	Q2	Q3	Q4	Q5	*P* for trend
**Total**						
Prediabetes	1 (reference)	1.37 (1.09–1.71)	1.35 (1.07–1.70)	1.36 (1.07–1.74)	1.15 (0.89–1.49)	0.48
Diabetes	1 (reference)	2.02 (1.44–2.82)	2.49 (1.79–3.47)	3.56 (2.56–4.96)	5.08 (3.65–7.07)	<0.0001
Dysglycemia	1 (reference)	1.55 (1.26–1.89)	1.66 (1.36–2.04)	1.96 (1.58–2.42)	2.08 (1.67–2.58)	<0.0001
**Men**						
Prediabetes	1 (reference)	1.29 (0.89–1.87)	1.45 (1.01–2.09)	1.45 (0.97–2.15)	1.53 (1.02–2.30)	0.0482
Diabetes	1 (reference)	2.05 (1.22–3.44)	3.47 (2.13–5.64)	3.68 (2.22–6.09)	6.31 (3.83–10.40)	<0.0001
Dysglycemia	1 (reference)	1.51 (1.10–2.09)	2.04 (1.50–2.79)	2.13 (1.52–2.97)	2.89 (2.06–4.05)	<0.0001
**Women**						
Prediabetes	1 (reference)	1.45 (1.09–1.93)	1.26 (0.93–1.72)	1.25 (0.92–1.71)	0.99 (0.71–1.38)	0.45
Diabetes	1 (reference)	1.62 (1.03–2.54)	2.17 (1.38–3.42)	3.28 (2.12–5.07)	4.38 (2.82–6.81)	<0.0001
Dysglycemia	1 (reference)	1.52 (1.17–1.97)	1.50 (1.14–1.98)	1.73 (1.31–2.27)	1.70 (1.28–2.25)	0.0008

a adjusted for age, sex, BMI, current smoking (yes or no), current alcohol consumption (yes or no) and family history of diabetes (yes or no).

Abbreviations: OR, odds ratio; CI, confidence interval.

In total population, the risk of having prediabetes significantly increased in Q2, Q3 and Q4 compared with Q1, but didn't in Q5. The risks of having diabetes or dysglycemia increased progressively across the lowest to highest quintile after adjustment for age, sex, BMI, current smoking and drinking status, as well as family history of diabetes.

At sex-specific analyses, compared with the lowest quintile, higher EZSCAN values of Q3 and Q5 in men, while of Q2 in women showed increased prevalence of prediabetes. For risk of having diabetes, the highest quintile had an OR of 6.31 (95% CI 3.83–10.40) in men and 4.38 (95% CI 2.82–6.81) in women compared with the lowest quintile. The similar significance was presented in the risk of having dysglycemia.

### The diagnostic performance of EZSCAN value to detect dysglycemia

The median (interquartile range) of EZSCAN value in total population was 42% (27–52%), and was a little higher in women than men [42% (27–52%) *vs.* 40% (27–51%), *P* value<0.0001].

**Figure 1:**
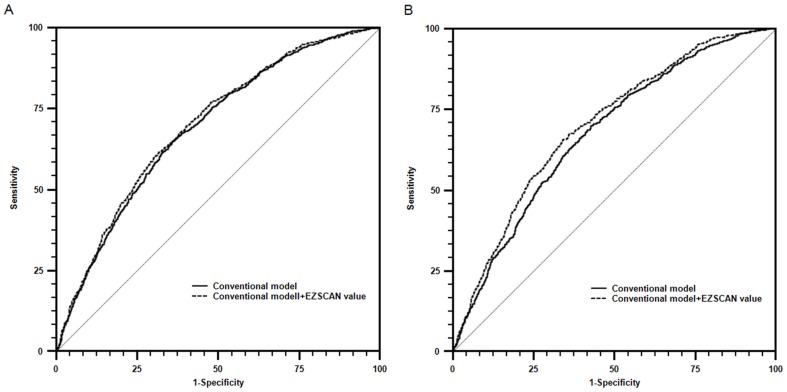
ROCs to detect dysglycemia (A) and diabetes (B). AUCs (95% CIs) in A were 0.689 (0.674–0.704) for conventional model and 0.697 (0.682–0.712) when EZSCAN value was added (*P* = 0.01). AUCs (95% CIs) in B were 0.676 (0.660–0.691) for conventional model and 0.700 (0.685–0.715) when EZSCAN value was added (*P*<0.0001). Age, BMI, family history of diabetes, women who delivered a giant baby or who were diagnosed with GDM, history of CVD, systolic blood pressure, diastolic blood pressure, HDL-C and TG were considered into conventional model.

Figure 1 (A) shows that the AUC for conventional model was 0.689 (95% CI 0.674–0.704) to detect dysglycemia, and was slightly increased to 0.697 (0.682–0.712) when EZSCAN value was added (*P* = 0.01). Figure 1 (B) shows that the AUC for conventional model was 0.676 (0.661–0.691) to detect diabetes, and was slightly increased to 0.700 (0.685–0.715) when EZSCAN value was added (*P*<0.0001).

Table 3 shows the sensitivities and specificities for detecting dysglycemia with EZSCAN values of 25%, 50%, 75% (each one-quarter increase from 0 to 100%), as well as 30% (threshold with sensitivities of more than 70%). The optimal cut-off point for EZSCAN to detect dysglycemia was 30%, not only in general analyses but also in sex-specific analyses. For detecting diabetes, sensitivities improved to 80.9% (95% CI 78.3–83.3%) in total population, 78.0% (73.8–81.7%) in men and 83.2% (79.8–86.2%) in women respectively. The results were similar in subjects without prior known diabetes (data not shown).

**Table 3:** Sensitivities and specificities to detect dysglycemia with different thresholds of EZSCAN value.

	Total			Men			Women		
EZSCAN value (%)	Numbers (cases)	Sensitivity (%)	Specificity (%)	Numbers (cases)	Sensitivity (%)	Specificity (%)	Numbers (cases)	Sensitivity (%)	Specificity (%)
**Dysglycemia**									
≥25	5127 (2161)	94.2 (93.1–95.1)	16.0 (14.8–17.2)	2050 (866)	93.7 (91.9–95.2)	16.6 (14.7–18.6)	3070 (1288)	94.5 (93.1–95.6)	15.6 (14.1–17.2)
≥30	3564 (1670)	72.8 (70.9–74.6)	46.3 (44.7–48.0)	1357 (650)	70.3 (67.3–73.3)	50.2 (47.5–52.8)	2201 (1014)	74.4 (72.0–76.7)	43.8 (41.6–45.9)
≥50	2054 (1055)	46.0 (43.9–48.1)	71.7 (70.2–73.2)	767 (403)	43.6 (40.4–46.9)	74.3 (72.0–76.6)	1285 (650)	47.7 (45.0–50.4)	69.9 (67.9–71.9)
≥75	54 (31)	1.4 (0.9–1.9)	99.3 (99.0–99.6)	23 (12)	1.3 (0.7–2.3)	99.2 (98.6–99.6)	31 (19)	1.4 (0.9–2.2)	99.4 (99.0–99.7)
**Diabetes**									
≥25	5127 (993)	97.6 (96.5–98.4)	14.0 (13.0–15.0)	2050 (438)	97.3 (95.3–98.5)	14.8 (13.3–16.5)	3070 (548)	97.9 (96.2–98.8)	13.5 (12.2–14.8)
≥30	3564 (823)	80.9 (78.3–83.3)	43.0 (41.6–44.4)	1357 (351)	78.0 (73.8–81.7)	46.9 (44.6–49.1)	2201 (466)	83.2 (79.8–86.2)	40.5 (38.7–42.3)
≥50	2054 (586)	57.6 (54.5–60.7)	69.5 (68.1–70.8)	767 (235)	52.2 (47.5–56.9)	71.9 (69.8–73.9)	1285 (349)	62.3 (58.1–66.3)	67.9 (66.1–69.6)
≥75	54 (23)	2.3 (1.5–3.4)	99.4 (99.1–99.6)	23 (10)	2.2 (1.1–4.2)	99.3 (98.8–99.6)	31 (13)	2.3 (1.3–4.0)	99.4 (99.0–99.6)
**Subgroup** a									
≥25	4882 (973)	97.8 (96.6–98.6)	12.0 (11.0–13.0)	1979 (432)	97.7 (95.7–98.8)	12.7 (11.2–14.4)	2903 (541)	97.8 (96.1–98.8)	11.5 (10.3–12.8)
≥30	3458 (811)	81.5 (78.9–83.8)	40.4 (39.0–41.9)	1335 (348)	78.7 (74.6–82.4)	44.3 (42.0–46.7)	2123 (463)	83.7 (80.3–86.7)	37.8 (36.0–39.7)
≥50	1994 (580)	58.3 (55.2–61.4)	68.2 (66.8–69.5)	754 (234)	52.9 (48.2–57.7)	70.7 (68.5–72.8)	1240 (346)	62.6 (58.4–66.6)	66.5 (64.7–68.3)
≥75	54 (23)	2.3 (1.5–3.5)	99.3 (99.0–99.5)	23 (10)	2.3 (1.2–4.3)	99.3 (98.7–99.6)	31 (13)	2.4 (1.3–4.1)	99.3 (98.9–99.6)

Values in parentheses are 95% confidence intervals.

a Subgroup analyses were done in individuals with high risk of developing diabetes [age ≥45 years or combined BMI ≥24 kg/m^2^ and family history of diabetes, or women delivered a giant baby or with GDM, or history of CVD, or hypertension (blood pressure ≥140/90 mmHg or on therapy for hypertension), or hyperlipmia (HDL-C<0.90 mmol/L and/or TG>2.82 mmol/L)].

### The diagnostic performance of EZSCAN value to detect diabetes in individuals with high risk of developing diabetes

We did a subgroup analysis of 5, 436 participants (2, 214 men and 3, 222 women) with high risk of developing diabetes, which included age ≥45 years or BMI ≥24 kg/m^2^ combined with one or more additional risk factors: 1) family history of diabetes, 2) women delivered a giant baby or with GDM, 3) history of CVD, 4) hypertension (blood pressure ≥140/90 mmHg or on therapy for hypertension), 5) hyperlipmia (HDL-C<0.90 mmol/L and/or TG>2.82 mmol/L) [17], [19] (Table 3). The mean (± SD) age of this subgroup was 59.4 (±9.4) years and the median (interquartile range) EZSCAN value was 42% (27–52%). The age was not significantly different in both sex (*P* = 0.63), while the EZSCAN value was higher in women than men [44% (27–52%) *vs.* 41% (27–52%), *P* = 0.0003]. In the subgroup analysis, an EZSCAN threshold of 30% provided slightly increased sensitivity of 81.5% (95% CI 78.9–83.8%) compared with the general analysis. The results were similar in sex-specific analyses.

### Assessment of potential risk factors for autonomic dysfunction

We next used EZSCAN value ≥50%, which was recommended as important sudomotor dysfunction by Impeto Medical [7], [8], [15], to study the risk factors of autonomic dysfunction (Table 4). After adjusted for the confounding factors (also including long-term uses of beta-blockers or antihypertensive 1, 4-dihydropyridines which might impact chloride concentration or uses of antidiabetic drugs), it was revealed that a significantly higher risk of having autonomic dysfunction was associated with female sex, older age, overweight and obesity. HDL-C level (per SD increase) was associated with a 9% lower risk of having autonomic dysfunction (adjusted OR = 0.91; 95% CI 0.85–0.97; *P* = 0.004). No significant relations between elevated systolic blood pressure, diastolic blood pressure, triglycerides and increased prevalence of autonomic dysfunction were detected.

**Table 4:** Multivariable-adjusted odds ratios for having autonomic dysfunction (EZSCAN value ≥50%).

Variable	SD	Unadjusted OR (95%CI)	*P* value	Adjusted OR (95%CI)^a^	*P* value
Female sex	-	1.21 (1.08–1.35)	0.0009	1.42 (1.25–1.62)	<0.0001
Age, per 10-yr increment	-	2.47 (2.32–2.63)	<0.0001	2.58 (2.38–2.80)	<0.0001
Overweight^b^	-	1.44 (1.27–1.63)	<0.0001	1.41 (1.22–1.63)	<0.0001
Obesity^c^	-	2.62 (2.25–3.05)	<0.0001	2.54 (2.12–3.05)	<0.0001
Systolic blood pressure, per increase of 10 mmHg	-	1.21 (1.18–1.24)	<0.0001	0.96 (0.91–1.00)	0.05
Diastolic blood pressure, per increase of 10 mmHg	-	0.97 (0.92–1.02)	0.27	1.02 (0.94–1.11)	0.59
TG, per increase of 1 SD (mmol/L)	1.47	1.11 (1.05–1.18)	0.0002	1.03 (0.96–1.09)	0.44
HDL-C, per increase of 1 SD (mmol/L)	0.32	0.91 (0.86–0.96)	0.001	0.91 (0.85–0.97)	0.004
FBG, per increase of 1 SD (mmol/L)	1.6	1.39 (1.31–1.47)	<0.0001	0.96 (0.86–1.08)	0.49
PBG, per increase of 1SD (mmol/L)	4.3	1.57 (1.49–1.67)	<0.0001	1.19 (1.07–1.31)	0.0008
HbA1c, per increase of 1 SD (%)	0.9	1.48 (1.39–1.57)	<0.0001	1.22 (1.09–1.36)	0.0004
Duration of diabetes^d^, per 5-yr increment	-	1.36 (1.11–1.67)	0.004	1.30 (1.01–1.67)	0.04

a Odds ratios were calculated with the use of multinomial logit models. All co-variables listed were included in the model simultaneously. Long-term uses of beta-blockers or antihypertensive 1, 4-dihydropyridines, uses of insulin or oral antidiabetic drugs were also put into the adjustment.

b Overweight was defined as a BMI between 24.0 kg/m^2^ and 27.9 kg/m^2^.

c Obesity was defined as a BMI of 28.0 kg/m^2^ or more. These two variables were all compared with normal BMI (<24.0 kg/m^2^) [19].

d analysis carried out only in diabetics.

For the glucose parameters, per SD increase of PBG and HbA1c levels displayed higher risks of having autonomic dysfunction. The adjusted ORs (95% CIs) were 1.19 (1.07–1.31) and 1.22 (1.09–1.36) respectively. The odds ratio was increased with per SD increase of FBG in unadjusted model (OR = 1.39; 95% CI 1.31–1.47; *P*<0.0001), but substantially decreased to 0.96 (95% CI 0.86–1.08; *P* = 0.49) after fully adjusted. Among diabetics with the information about disease duration (n = 432), longer duration of diabetes (per 5 years increment) was associated with higher risk of having autonomic dysfunction independently (unadjusted OR = 1.36; 95% CI 1.11–1.67; *P* = 0.004; adjusted OR = 1.30; 95% CI 1.01–1.67; *P* = 0.04).

## Discussion

In the present study, we investigated the association between different glucose status and autonomic dysfunction assessed by EZSCAN device. More importantly, the discriminatory values of EZSCAN measurements for the risk of prediabetes and diabetes were established in a population-based study among 5, 824 Chinese men and women. To our knowledge, this is the first large scale population-based study evaluating the autonomic dysfunction and its risk factors by EZSCAN.

According to our results, EZSCAN values were increased in individuals with prediabetes and diabetes compared with NGT group, indicating that autonomic function might degenerate early in prediabetes, and aggravate in diabetes. This result was in accordance with several other previous case-control studies carried out in European and Indian subjects [7], [8]. Furthermore, compared with the lowest quintile, high EZSCAN value significantly increased the risk of having dysglycemia regardless of sex after adjustment for confounding factors.

Based on the intensive association between autonomic dysfunction and dysglycemia, we next explored the usefulness of this autonomic test in early identification for dysglycemia. The receiver operating characteristic curves showed that EZSCAN value improved the AUCs for detection of dysglycemia or diabetes beyond the contribution of conventional risk factors by 0.8% and 2.4%. However, it should be emphasized that the AUCs were less than 0.7 and the increase were mild. The results indicated that EZSCAN was not suitable to perform as a diagnostic tool.

Accordingly, in order to obtain a better screening and prediction power for dysglycemia, we determined a cut-off point of 30% with which the sensitivities were more than 70%. The sensitivity of this threshold further increased to more than 80% for detecting diabetes in subjects with high risk of developing diabetes. This growth trend was also demonstrated in sex-specific analyses. Thus we strongly proposed to use the cut-off point 30% for the large-scale screening of dysglycemia, including prediabetes and diabetes in middle-aged and elderly population, and use the same cut-off point in men and women. Subjects with EZSCAN value ≥30% are considered as individuals at a high risk of having dysglycemia. They should be further confirmed by glycemia tests.

Besides, the sensitivity and specificity using cut-off point 50% in our study were lower than the reports from French study [7] and Indian study [8]. This could be explained by different study design. The previous French study was a case-control analysis, comparing 133 diabetics at the mean age of 58.9 years with 41 healthy controls at the mean age of 25.5 years, and the Indian study included 24 diagnosed diabetics, 30 IGT subjects and 158 NGT subjects. It should be noted that a variety of elements, such as age, sex and BMI, are all strong risk factors for autonomic dysfunction. However, in the present study, the power of EZSCAN value to differentiate subjects with dysglycemia as well as diabetes from NGT subjects was independent of these confounders.

Another Chinese study [9] also showed low sensitivity when using 50% as threshold and selected 40% for the diagnosis of diabetes eventually. Combined with our study, it was likely that lower threshold was suitable for Chinese in order to achieve reasonable sensitivity and specificity compared with French and Indian studies. As a population-based study, we suggested to adopt our cut-off compared to the small-size case-control study.

Consistent with previous studies, we have clearly shown the association between autonomic dysfunction with some conventional risk factors, including female sex, elder age, obesity, hyperglycemia and lower level of HDL-C [20]. The present study also supported the important association between autonomic dysfunction with postprandial glycemia and HbA1c, whereas much weaker association with fasting glycemia by regression models. The reason might be complicated and unclear. It is suggested that impaired fasting glucose and impaired glucose tolerance may contribute to autonomic dysfunction in different way. On one hand, consistent with Watkins et al [21], our results also demonstrated that the relationship between FBG and autonomic function was largely mediated by the co-occurrence of high blood pressure, elevated BMI, and old age. It was considered that elevated fasting glucose may be less directly related to impaired autonomic control. On the other hand, impaired glucose tolerance appears a predominant small-fiber neuropathic involvement [22], which sudomotor dysfunction belongs to. Besides, general decrease in the amplitudes of sympathetic skin responses indicated that sudomotor fibers tended to be affected earlier in the group with postprandial hyperglycemia [3]. Therefore, OGTT should remain an important investigation into the work-up of patients with neuropathy [23].

We also demonstrated longer duration of diabetes was associated with high risk of having autonomic dysfunction defined as EZSCAN value ≥50%, independently of other confounding factors. Chronic glycemic exposure plays the key role in the development and progression of diabetic neuropathies. Experimental data implicate a number of pathogenic pathways that may impact autonomic function in diabetics including: formation of advanced glycation end products, increased oxidative/nitrosative stress with increased free radical production, activation of the polyol and protein kinase C pathways, activation of polyADP ribosylation, and activation of genes involved in neuronal damage [24], [25], [26]. Several mutual proinflammatory cytokines and adipocytokines also act on the pathogenesis of autonomic neuropathy and diabetes, such as interleukin 6, C-reactive protein, leptin and adiponectin [27]. However, Dyck et al [28] suggested that a combination of age at onset of diabetes, HbA1c level and duration of diabetes predicted complications better than single components of chronic glycemic exposure (the degree or duration of hyperglycemia).

The strengths of the present study lie in its large-scale, population-based design and inclusion of participants spanning a wide age range of 40–92 yr. Although some other studies have suggested EZSCAN thresholds, their sample sizes were much smaller [7], [8], [29]. Our study also provided useful information on the relations between EZSCAN value and diabetes, or other metabolic risk factors, whereas other studies were only case-control design with limited confounding factors.

However, several limitations of our study worth mention. First, all findings that we presented here were derived from a cross-sectional investigation. Replication of the current results in separate populations, especially in long-term data, is required to confirm the usefulness of EZSCAN in detecting and predicting the risk of having prediabetes and diabetes. Second, the study was done in a population aged 40 years and older, future studies in different age group or ethnicity should be carried out to evaluate the performance of the EZSCAN. Third, risk factors such as physical activity, history of endocrine and metabolic disease, and previous test of glycemia could not be considered in the present study due to lack of detailed information. Fourth, we tested sudomotor function only once for each person, thus the reproducibility of EZSCAN could not be examined which had checked by the previous studies [7], [15]. Fifth, subjects with abnormal EZSCAN value have not re-examined by QSART.

## Conclusions

Conclusively, the present population-based study suggests the EZSCAN device as a screening measurement for middle-aged and elderly individuals at high risk of diabetes. We recommended that subjects with EZSCAN ≥30% should be further evaluated by OGTT, especially when suffering from other conventional risk factors. It was worth mentioning that even if EZSCAN significantly improves AUC compared with its exclusive use of risk factors for diabetes detection, the magnitude of this increase is of limited clinical relevance. Thus the clinical application value of EZSCAN is needed to be confirmed in future studies.
